# Increased Interleukin-6 Levels in the Astrocyte-Derived Exosomes of Sporadic Amyotrophic Lateral Sclerosis Patients

**DOI:** 10.3389/fnins.2019.00574

**Published:** 2019-06-05

**Authors:** Yong Chen, Kailin Xia, Lu Chen, Dongsheng Fan

**Affiliations:** ^1^Department of Neurology, Peking University Third Hospital, Beijing, China; ^2^Key Laboratory for Neuroscience, Ministry of Education/National Health Commission, Peking University, Beijing, China

**Keywords:** amyotrophic lateral sclerosis, astrocytes, exosomes, disease progress, interleukin-6

## Abstract

Neuroinflammation plays an important role in amyotrophic lateral sclerosis (ALS) pathogenesis. However, it is difficult to evaluate inflammation of the central nervous system (CNS) or the relationship between neuroinflammation and disease progression in ALS patients. Recent advances in the field of exosomes and CNS-derived exosomes extraction technology provide the possibility of measuring the inflammatory status in the CNS without brain biopsy. In this pilot study, we extracted astrocyte-derived exosomes from the plasma of sporadic ALS patients and age-, sex-matched healthy controls and determined Interleukin-6 (IL-6) levels by an enzyme-linked immunosorbent assay (ELISA). The IL-6 levels in astrocyte-derived exosomes were increased in sALS patients and positively associated with the rate of disease progression. However, the association between IL-6 levels and disease progression rate was limited to patients whose disease duration were less than 12 months. These data suggest an increased inflammatory cascade in the CNS of sALS patients. Our pilot study demonstrates that CNS-derived exosomes could be useful to reveal neuroinflammation of the CNS in ALS patients.

## Introduction

Amyotrophic lateral sclerosis (ALS) is a rare, progressive neurodegenerative disease that affects upper and lower motor neurons and leads to fatal paralysis (Brown and Al-Chalabi, 2017). Ultimately, most ALS patients die within 3–5 years after disease onset due to respiratory failure. Approximately 90–95% of ALS cases are the sporadic type (sALS), and the remaining cases are the familial type (fALS). To date, more than 20 genes that cause fALS and sALS have been identified (Brown and Al-Chalabi, 2017). Scientific advances in genetic studies in the ALS field have improved our understanding of ALS pathogenesis. However, the exact etiology and pathogenesis of ALS are still unknown. As a result, there is no effective treatment for the disease. Riluzole and edaravone are the only two approved drugs for the treatment of ALS, and they solely delay disease progression for several months (Kumar et al., 2016; Rothstein, 2017).

Numerous intrinsic and extrinsic factors are involved in ALS motor neuron degeneration. One possible factor involved in motor neuron degeneration in ALS is neuroinflammation. Accumulating evidence indicates that ALS patients have chronic inflammation, as demonstrated by activated microglia and astrocytes, as well as infiltration of peripheral monocytes and lymphocytes into the CNS (Zhao et al., 2013; Liu and Wang, 2017). Increased serum/plasma and CSF levels of some cytokines, such as tumor necrosis factor-alpha (TNF-α), interleukin-6 (IL-6), IL-8, and interferon-beta (IFN-β), have been detected in ALS patients when compared to controls (Ono et al., 2001; Mitchell et al., 2009; Fiala et al., 2010; Mitchell et al., 2010; Italiani et al., 2014; Ehrhart et al., 2015; Liu et al., 2015; Hu et al., 2017). Beyond demonstrating ongoing inflammatory processes in ALS patients, these inflammatory biomarkers could also be used as diagnostic and prognostic biomarkers for clinical use because they have been reported to distinguish ALS from healthy or disease controls (Vu and Bowser, 2017; Gonzalez-Garza et al., 2018) and to predict the disease prognosis (Su et al., 2013; Liu et al., 2015). Activated microglia and astrocytes in the CNS play a vital role in the neuroinflammation process in ALS patients; however, the determination of the inflammatory biomarkers in serum/plasma and CSF only indirectly reflects the status of the CNS. Recently, scientific advances in the field of exosomes and CNS-derived exosome extraction technology have provided the possibility of measuring the inflammatory status in the CNS without brain biopsy.

Exosomes are approximately 30–100 nm extracellular vesicles with lipid bilayer membranes that are secreted by almost all types of cells, including neurons, microglia and astrocytes (Raposo and Stoorvogel, 2013; Yanez-Mo et al., 2015). Exosomes contain proteins, lipids and RNA and transfer them between cells. Therefore, exosomes play an important role in intercellular communication. Moreover, different cell types can secrete exosomes with different biomarkers, which could help to identify the exosome source (Beninson and Fleshner, 2014). Due to their specific characteristics, exosomes have attracted large amounts of attention in various studies ranging from mechanistic analyses to clinical research (Jarmalaviciute and Pivoriunas, 2016; Goh et al., 2017). In addition, exosomes can cross the blood-brain barrier (BBB) from both directions. As a result, CNS-derived exosomes can be detected in the blood and may help to reveal the pathophysiology of brain diseases without the use of brain biopsy and CSF analysis (Mustapic et al., 2017). In recent studies, several strategies to extract CNS-derived exosomes from peripheral blood have been reported (Mustapic et al., 2017; Kuwano et al., 2018). However, CNS-derived exosome-based studies focusing on ALS have not been previously reported.

Based on the above information, we hypothesized that inflammatory biomarkers in astrocyte-derived exosomes (ADEs) may increase and may be associated with clinical features in ALS patients. In this pilot study, we extracted ADEs from the plasma of sporadic ALS patients and age-, sex-matched healthy controls to determine the IL-6 levels in ADEs and, ultimately, we detected increased IL-6 levels in ADEs of sALS patients, which were positively associated with the rate of disease progression.

## Participants and Methods

### Participants

This study was approved by the Ethics Committee of the Perking University Third Hospital, Beijing, China. All ALS patients and age-, sex-matched healthy control individuals signed the informed consent before peripheral blood samples were drawn. Patients and controls were recruited from the Department of Neurology of Perking University Third Hospital. Clinically definite and probable sALS patients were diagnosed based on the EI Escorial revised criteria (Brooks et al., 2000) and further evaluated by the revised ALS functional rating scale (ALSFRS-R) (Cedarbaum et al., 1999). The rate of disease progression (ΔFS) was calculated as follows: ΔFS = (48 -ALSFRS-R at “time of diagnosis”)/duration from onset to diagnosis (month) (Kimura et al., 2006).

### Plasma Sampling in ALS Patients and Controls

Samples containing two milliliters of peripheral blood from ALS patients and healthy control individuals were collected into EDTA tubes. To extract plasma, blood samples were centrifuged at 1500 *g* for 10 min to remove blood cells. Then, the supernatant was subjected to another centrifugation at 2500 *g* for 20 min to remove the platelets and cell debris. Finally, the plasma was stored at −80°C until use.

### Extraction of ADEs From Plasma

The method to extract the ADEs from plasma was modified from a previously published article (Mustapic et al., 2017). Briefly, 0.25 ml plasma was incubated with 0.2 μl thromboplastin (System Biosciences, Mountain View, CA, United States) for 5 min. Then, 298 μl calcium- and magnesium-free Dulbecco’s Balanced Salt Solution (DBS^–2^) was added with protease inhibitor cocktail (Roche, Indianapolis, IN) and phosphatase inhibitor cocktail (Thermo Fisher Scientific), followed by centrifugation at 10,000 rpm for 5 min at 4°C. The supernatants were harvested, followed by addition of 126 μl per tube of ExoQuick (System Biosciences, Mountain View, CA, United States). After a second centrifugation at 1500 g for 30 min at 4°C, total exosomes were harvested by removing the supernatant. To enrich ADEs, total exosomes were resuspended in 250 μl of ddH_2_O with protease inhibitor cocktail and phosphatase inhibitor cocktail and incubated for at least 120 min at 4°C. Then, 1.5 μg biotinylated mouse anti-human glutamine aspartate transporter (ACSA-1) antibody (Miltenyi Biotec, Auburn, CA, United States) in 50 ml of 3% bovine serum albumin (BSA; 1:3.33 dilution of Blocker BSA 10% solution in DBS^–2^; Thermo Fisher Scientific) was added per tube and mixed for 60 min at room temperature, followed by the addition of 10 μl streptavidin-agarose Ultralink resin (Thermo Fisher Scientific) in 40 ml 3% BSA and incubation with mixing for another 20 min at room temperature. After centrifugation at 400 *g* for 10 min at 4°C, the supernatant was removed, and each pellet was suspended in 200 μl cold 0.1 M glycine-HCl (pH = 3.0) by gentle mixing for 10 s and centrifugation at 4,500 *g* for 5 min. The supernatants were then harvested, and 25 μl of 3% BSA and 15 μl of 1 M Tris–HCl (pH = 8.0) were added. Finally, 260 μl mammalian protein extraction reagent (M-PER, Thermo Fisher Scientific) was added, and the solution was mixed. The resultant 0.5 ml lysates of ADEs were stored at −80°C. Evidence for enrichment of exosomes from neural sources in plasma has been demonstrated previously (Mustapic et al., 2017).

### Measurement of IL-6 Levels in ADEs and Plasma

Astrocyte-derived exosome proteins were quantified using a single-plex high-sensitivity and high-dynamic-range ELISA for IL-6 (Rockville, MD, United States Cat# K151AKC) (Chaturvedi et al., 2015) and by using enzyme-linked immunosorbent assay (ELISA) kits for the tetra-spanning exosome marker CD81 (Cusabio Technology, Wuhan, China), according to the suppliers’ directions. The mean value for all determinations of CD81 in each assay group was set at 1.00, and the relative values for each sample were used to normalize their recovery. The plasma IL-6 levels in both groups were also measured. The protein levels were measured by board-certified laboratory technicians who were blinded to the clinical information.

To ensure the specificity of the tests, negative control groups were set up in this study. In the negative control group one, the biotinylated anti-ACSA-1 antibody was replaced with 3% BSA. In the negative control group two, the total exosomes solution resuspended from ExoQuick pellet was replaced by ddH2O.

### Statistical Analyses

Data are presented as numbers, means and standard deviations, or medians (interquartile range, IQR) as appropriate. Normal distributions of datasets were assessed by the Shapiro–Wilks test. Unpaired Student *t*-tests, χ^2^ test or one-way ANOVA, followed by Tukey analysis, were used to examine differences between groups. Pearson’s correlation was used for statistical correlation analysis. The differences between groups were considered significant if the *p*-value was less than 0.05 (two-tailed). All statistical analyses and graphs were performed using GraphPad Prism 6 (GraphPad Software Inc., San Diego, United States).

## Results

**FIGURE 1:**
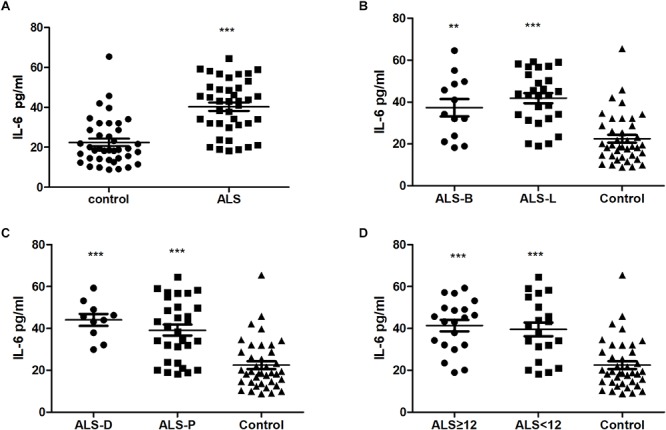
Comparison IL-6 levels in ADEs from plasma of ALS and healthy subjects. Panels **(A–D)** show the levels of IL-6 in ADEs of **(A)** ALS patients and controls; **(B)** ALS patients with bulbar (ALS-B) or limb onset (ALS-L) and controls; **(C)** definite ALS (ALS-D) or probable ALS (ALS-P) and controls; **(D)** ALS duration ≥ 12 months (ALS ≥ 12) or <12 months (ALS < 12) and controls. ^∗∗^, ^∗∗∗^ indicate *p* < 0.01 and *p* < 0.001, respectively, compared with controls.

In this pilot study, 40 ALS patients and 39 healthy controls were recruited. The detailed clinical information for these two groups are summarized in Table 1. The ALS patients and controls were comparable, as there was no difference in age or sex ratio between the two groups. Of the 40 ALS patients, 12 were bulbar onset and 28 were limber onset; 10 ALS cases were diagnosed as definite, and the remainder were probable. The median delay of diagnosis for all patients was 9.23 months. The mean ALSFRS-R score for the patients was 39.83 ± 1.08, and the median disease progression rate was 0.56. The extracted ADEs were validated by western blot. The result showed that the ADEs were positive for CD63, but negative for calnexin (Supplementary Figure S1). The ADEs were also verified by transmission electron microscope (Supplementary Figure S2). In the CD81 and IL-6 test, the negative control group one and two were all at background levels. The CD81-normalized levels of IL-6 in ADEs were significantly higher in ALS patients (40.40 ± 2.11 pg/ml) than in controls (22.45 ± 1.90 pg/ml) (Figure 1A). However, among 40 ALS patients and 39 healthy controls, the IL-6 was detectable only in 12 controls and 15 ALS patients. There was no difference in detection rate between the two groups. The plasma IL-6 levels ranged from 0.13 to 4.58 pg/mL in controls and 0.39 to 15.69 pg/ml in ALS patients (Supplementary Figure S3A). There was no difference in plasma IL-6 levels between controls and ALS patients (*p* = 0.3614) and there was no correlation between IL-6 levels in plasma and ADEs (*r* = 0.3384, *p* = 0.2173 for ALS group; *r* = −0.2657, *p* = 0.4038 for control group; Supplementary Figures S3B,C).

**TABLE 1:** Characteristics of ALS patients and healthy controls.

	**Control**	**ALS**	***p***
Cases (male/female)	39 (25/14)	40 (26/14)	1
Age (mean ± SE)	55.74 ± 1.32	54.35 ± 2.02	0.57
Onset site: bulbar/limb	NA	12/28	NA
Diagnosis delay (months)	NA	9.23 (9.68)	NA
Definite/probable	NA	10/30	NA
ALSFRS-R	NA	39.83 ± 1.08	NA
ΔFS	NA	0.56 (0.71)	NA
IL-6 (pg/ml)	22.45 ± 1.90	40.40 ± 2.11	<0.001

*Diagnosis delay, interval from the initial symptoms to diagnosis; ALSFRS-R, revised amyotrophic lateral sclerosis functional rating scale; ΔFS, disease progression rate.*

The ALS patients were further divided into subgroups according to the following: onset site: bulbar onset (ALS-B) or limber onset (ALS-L); diagnosis level: definite (ALS-D) or probable (ALS-P); and disease duration: less than 12 months (ALS < 12) or greater than or equal to 12 months (ALS ≥ 12). As shown in Figures 1B–D, compared with the control group, the levels of IL-6 in ADEs were increased in all ALS subgroups. However, there was no difference between the ALS subgroups.

**FIGURE 2:**
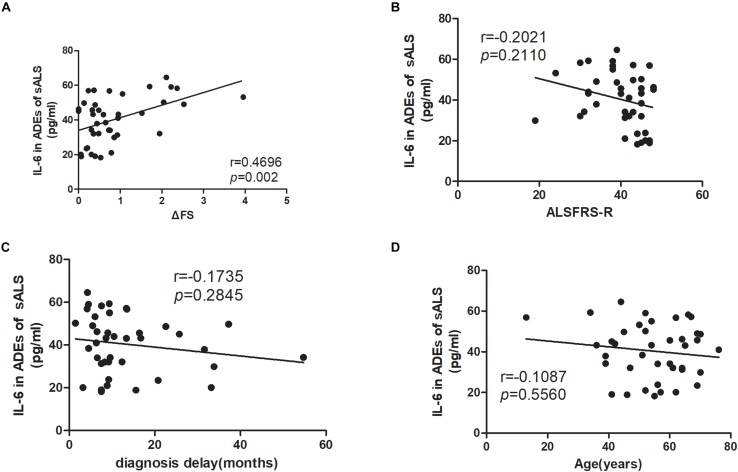
Correlations between IL-6 levels in ADEs of ALS patients with the disease progression rate, ALSFRS-R score, diagnosis delay and patient age. **(A)** shows that the IL-6 levels in ADEs of ALS patients positively correlate with the disease progression rate. However, the IL-6 levels in ADEs of ALS patients do not correlate with the ALSFRS-R score **(B)**, diagnosis delay **(C),** and patient age **(D)**.

**FIGURE 3:**
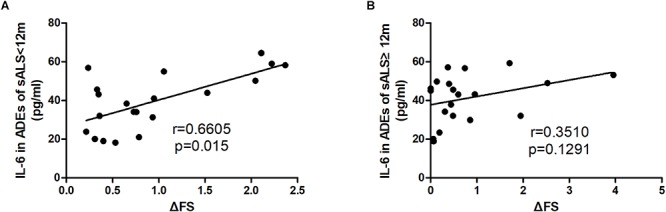
Correlations between IL-6 levels in ADEs of ALS subgroups with the disease progression rate. ALS patients were divided into two groups according to the disease duration. **(A)** The IL-6 levels in ADEs of the ALS < 12 group positively correlate with the disease progression rate. However, the Il-6 levels in ADEs of the ALS12 group do not correlate with the disease progression rate **(B)**.

The correlations of the levels of IL-6 in ADEs with clinical parameters are shown in Figure 2. The IL-6 levels correlated positively with the disease progression rate (*r* = 0.4696, *p* = 0.002). However, IL-6 levels in the ADEs of ALS patients did not correlate with total ALSFRS-R scores (*r* = −0.2021, *p* = 0.2110), diagnosis delay (*r* = −0.1735, *p* = 0.2845) or patient age (*r* = −0.1087, *p* = 0.5560). In controls, IL-6 levels also did not correlate with age (data not shown). When the patients were separated into two groups according disease duration (ALS < 12 m or ALS ≥ 12 m), a positive correlation between IL-6 levels and disease progression was only verified in the ALS < 12 m group (*r* = 0.6605, *p* = 0.015) (Figure 3A) but not in the ALS ≥ 12 m group (*r* = 0.3510, *p* = 0.1291) (Figure 3B).

## Discussion

The present study demonstrated that the levels of IL-6 in ADEs of sALS patients were increased and positively associated with the rate of disease progression, especially in patients at an earlier disease stage. These data suggest that the inflammatory cascade is augmented in the CNS of sALS patients. Analysis of CNS-derived exosomes in peripheral blood has recently attracted immense attention. Numerous studies have demonstrated that CNS-derived exosomes could be helpful to understand the pathophysiology of brain disease and the identification of biomarkers (Abner et al., 2016; Winston et al., 2016; Goetzl et al., 2018; Ohmichi et al., 2018). However, to our knowledge, no studies have been reported on CNS-derived exosomes in ALS patients. Therefore, our pilot study is the first to demonstrate that CNS-derived exosomes could be useful to reveal the pathophysiology of CNS in ALS patients.

Several inflammatory biomarkers have been found to be linked to ALS. As a well-known cytokine, IL-6 has been extensively investigated in neurodegenerative disorders and associated with ALS in numerous studies (Sekizawa et al., 1998; Ehrhart et al., 2015; Lu et al., 2016; Blasco et al., 2017; Hu et al., 2017). However, the results are not consistent across all studies (Moreau et al., 2005; Tanaka et al., 2006). In addition, one study reported an increase in IL-6 levels at the late stage of disease (Lu et al., 2016), whereas another study reported that the levels of IL-6 were high at disease onset followed by a subsequent decline (Ehrhart et al., 2015). The plasma IL-6 levels were also measured in this study. However, the IL-6 was detectable in only 12 controls and 15 ALS patients and undetectable in most of the samples. Among the 12 controls and 15 ALS patients, the IL-6 levels were highly variable and no difference has been found between the two groups. The highly variable plasma IL-6 levels in our study and the contradictory results from previous studies indicate that the peripheral IL-6 levels may be influenced by complex factors. A recent study showed that the levels of IL-6 in blood could be influenced by aging and respiratory dysfunction in ALS (Pronto-Laborinho et al., 2019). Thus, determining the IL-6 levels in blood may not be a good way. CNS-derived exosomes could directly reflect the situation in the CNS, and peripheral factors might have little effect on cytokines in CNS-derived exosomes. Therefore, the measurement of IL-6 levels in CNS-derived exosomes, compared with blood or CSF, may be better to illuminate the actual role of IL-6 in ALS. Astrocytes have been reported play an important role in the pathogenesis of ALS, and the predominant CNS source of IL-6 is the activated astrocyte (Van Wagoner and Benveniste, 1999). Hence, in this pilot study, we chose to measure IL-6 levels in ADEs. Compared with the plasma IL-6 levels, the IL-6 levels in ADEs were relatively high and stable and the IL-6 levels in the ADEs didn’t correlate with age. Moreover, it was supposed that there may be connection between IL-6 levels in plasma and ADEs. However, no correlation had been found between two groups. All these results indicate that CNS-derived exosomes may be a promising object to help find biomarkers for ALS.

The important findings of our study were that IL-6 levels in ADEs increased in sALS patients and were positively associated with the rate of disease progression. These data suggest that the IL-6 in ADEs may be a candidate biomarker for ALS. However, neuroinflammation is a common phenomenon in almost all neurological disease. Therefore, it is believed that the IL-6 levels in ADEs probably increase in other neurological conditions. Actually, it has been reported that the IL-6 levels in ADEs increased in AD patients (Goetzl et al., 2018). Thus, the IL-6 levels in ADEs may not be suitable to help discriminate ALS from other neurological diseases. According to our study, measuring the IL-6 levels in ADEs may be helpful to reflect the neuroinflammation status and predict disease progression.

We could not determine the precise role of IL-6 in ALS patients because of its complex physiological functions. Increased IL-6 secretion could be a neuroprotective reaction against CNS damage or a pro-inflammatory agent (Spooren et al., 2011). However, most views consider IL-6 as a pro-inflammatory cytokine in ALS patients. The anti-IL-6 antibody, tocilizumab, has been proposed as a therapeutic drug for ALS (Fiala et al., 2013). Therefore, we speculate that the increase in IL-6 observed in this study was harmful to ALS patients. Our further analyses revealed that the levels of IL-6 did not differ between ALS subgroups, and the correlation between IL-6 and the rate of disease progression was only observed during the initial 12 months. These results indicated that IL-6 produced by astrocytes might be more important during the early stage of disease. However, our sample size was limited, and the results should therefore be confirmed in further studies.

## Conclusion

The present study demonstrated that the levels of IL-6 in ADEs of ALS patients were increased and positively associated with the rate of disease progression, especially in patients at an earlier disease stage. Our pilot study is the first to demonstrate that CNS-derived exosomes could be useful to reveal the pathophysiology of CNS in ALS patients.

## Data Availability

The raw data supporting the conclusions of this manuscript will be made available by the authors, without undue reservation, to any qualified researcher.

## Ethics Statement

This study was approved by the Ethics Committee of the Perking University Third Hospital, Beijing, China. All ALS patients and age-, sex-matched healthy control individuals signed the informed consent before peripheral blood samples were drawn.

## Author Contributions

DF conceived the study, provided the financial support, and responsible for the project management. DF and YC designed the study, responsible for preparing and revising the manuscript, and had key roles in the study. KX and LC took part in the design of the study and in sample collection, and undertook data checking.

## Conflict of Interest Statement

The authors declare that the research was conducted in the absence of any commercial or financial relationships that could be construed as a potential conflict of interest.

## References

[B1] Abner E. L. Jicha G. A. Shaw L. M. Trojanowski J. Q. Goetzl E. J. (2016). Plasma neuronal exosomal levels of Alzheimer’s disease biomarkers in normal aging. *Ann. Clin. Transl. Neurol.* 3 399–403. 10.1002/acn3.309 27231710 PMC4863753

[B2] Beninson L. A. Fleshner M. (2014). Exosomes: an emerging factor in stress-induced immunomodulation. *Semin. Immunol.* 26 394–401. 10.1016/j.smim.2013.12.001 24405946

[B3] Blasco H. Garcon G. Patin F. Veyrat-Durebex C. Boyer J. Devos D. (2017). Panel of oxidative stress and inflammatory biomarkers in ALS: a pilot study. *Can. J. Neurol. Sci.* 44 90–95. 10.1017/cjn.2016.284 27774920

[B4] Brooks B. R. Miller R. G. Swash M. Munsat T. L. World Federation of Neurology Research Group on Motor Neuron Diseases. (2000). El Escorial revisited: revised criteria for the diagnosis of amyotrophic lateral sclerosis, amyotroph lateral scler other motor. *Neuron Disord.* 1 293–299. 10.1080/146608200300079536 11464847

[B5] Brown R. H. Al-Chalabi A. (2017). Amyotrophic lateral sclerosis. *N. Engl. J. Med.* 377 162–172. 28700839 10.1056/NEJMra1603471

[B6] Cedarbaum J. M. Stambler N. Malta E. Fuller C. Hilt D. Thurmond B. (1999). The ALSFRS-R: a revised ALS functional rating scale that incorporates assessments of respiratory function. BDNF ALS study group (Phase III). *J. Neurol. Sci.* 169 13–21. 10.1016/s0022-510x(99)00210-5 10540002

[B7] Chaturvedi S. Siegel D. Wagner C. L. Park J. van de Velde H. Vermeulen J. (2015). Development and validation of panoptic Meso scale discovery assay to quantify total systemic interleukin-6. *Br. J. Clin. Pharmacol.* 80 687–697. 10.1111/bcp.12652 25847183 PMC4594705

[B8] Ehrhart J. Smith A. J. Kuzmin-Nichols N. Zesiewicz T. A. Jahan I. Shytle R. D. (2015). Humoral factors in ALS patients during disease progression. *J. Neuroinflammation* 12:127. 10.1186/s12974-015-0350-4 26126965 PMC4487852

[B9] Fiala M. Chattopadhay M. La Cava A. Tse E. Liu G. Lourenco E. (2010). IL-17A is increased in the serum and in spinal cord CD8 and mast cells of ALS patients. *J. Neuroinflammation* 7:76. 10.1186/1742-2094-7-76 21062492 PMC2992053

[B10] Fiala M. Mizwicki M. T. Weitzman R. Magpantay L. Nishimoto N. (2013). Tocilizumab infusion therapy normalizes inflammation in sporadic ALS patients. *Am. J. Neurodegener. Dis.* 2 129–139. 23844337 PMC3703125

[B11] Goetzl E. J. Schwartz J. B. Abner E. L. Jicha G. A. Kapogiannis D. (2018). High complement levels in astrocyte-derived exosomes of Alzheimer disease. *Ann. Neurol.* 83 544–552. 10.1002/ana.25172 29406582 PMC5867263

[B12] Goh W. J. Zou S. Ong W. Y. Torta F. Alexandra A. F. Schiffelers R. M. (2017). Bioinspired cell-derived nanovesicles versus exosomes as drug delivery systems: a cost-effective alternative. *Sci. Rep.* 7:14322. 10.1038/s41598-017-14725-x 29085024 PMC5662560

[B13] Gonzalez-Garza M. T. Martinez H. R. Cruz-Vega D. E. Hernandez-Torre M. Moreno-Cuevas J. E. (2018). Adipsin. MIP-1b, and IL-8 as CSF biomarker panels for ALS diagnosis. *Dis. Markers* 2018:3023826. 10.1155/2018/3023826 30405855 PMC6199888

[B14] Hu Y. Cao C. Qin X. Y. Yu Y. Yuan J. Zhao Y. (2017). Increased peripheral blood inflammatory cytokine levels in amyotrophic lateral sclerosis: a meta-analysis study. *Sci. Rep.* 7:9094. 10.1038/s41598-017-09097-1 28831083 PMC5567306

[B15] Italiani P. Carlesi C. Giungato P. Puxeddu I. Borroni B. Bossu P. (2014). Evaluating the levels of interleukin-1 family cytokines in sporadic amyotrophic lateral sclerosis. *J. Neuroinflammation* 11:94. 10.1186/1742-2094-11-94 24884937 PMC4039322

[B16] Jarmalaviciute A. Pivoriunas A. (2016). Exosomes as a potential novel therapeutic tools against neurodegenerative diseases. *Pharmacol. Res.* 113(Pt B), 816–822. 10.1016/j.phrs.2016.02.002 26855317

[B17] Kimura F. Fujimura C. Ishida S. Nakajima H. Furutama D. Uehara H. (2006). Progression rate of ALSFRS-R at time of diagnosis predicts survival time in ALS. *Neurology* 66 265–267. 10.1212/01.wnl.0000194316.91908.8a 16434671

[B18] Kumar V. Islam A. Hassan M. I. Ahmad F. (2016). Therapeutic progress in amyotrophic lateral sclerosis-beginning to learning. *Eur. J. Med. Chem.* 121 903–917. 10.1016/j.ejmech.2016.06.017 27372371

[B19] Kuwano N. Kato T. A. Mitsuhashi M. Sato-Kasai M. Shimokawa N. Hayakawa K. (2018). Neuron-related blood inflammatory markers as an objective evaluation tool for major depressive disorder: an exploratory pilot case-control study. *J. Affect Disord.* 240 88–98. 10.1016/j.jad.2018.07.040 30059939

[B20] Liu J. Gao L. Zang D. (2015). Elevated levels of IFN-gamma in CSF and serum of patients with amyotrophic lateral sclerosis. *PLoS One* 10:e0136937. 10.1371/journal.pone.0136937 26332465 PMC4557946

[B21] Liu J. Wang F. (2017). Role of neuroinflammation in amyotrophic lateral sclerosis: cellular mechanisms and therapeutic implications. *Front. Immunol.* 8:1005. 10.3389/fimmu.2017.01005 28871262 PMC5567007

[B22] Lu C. H. Allen K. Oei F. Leoni E. Kuhle J. Tree T. (2016). Systemic inflammatory response and neuromuscular involvement in amyotrophic lateral sclerosis. *Neurol. Neuroimmunol. Neuroinflamm.* 3:e244. 10.1212/NXI.0000000000000244 27308305 PMC4897985

[B23] Mitchell R. M. Freeman W. M. Randazzo W. T. Stephens H. E. Beard J. L. Simmons Z. (2009). A CSF biomarker panel for identification of patients with amyotrophic lateral sclerosis. *Neurology* 72 14–19. 10.1212/01.wnl.0000333251.36681.a5 18987350

[B24] Mitchell R. M. Simmons Z. Beard J. L. Stephens H. E. Connor J. R. (2010). Plasma biomarkers associated with ALS and their relationship to iron homeostasis. *Mus. Nerve* 42 95–103. 10.1002/mus.21625 20544912

[B25] Moreau C. Devos D. Brunaud-Danel V. Defebvre L. Perez T. Destee A. (2005). Elevated IL-6 and TNF-alpha levels in patients with ALS: inflammation or hypoxia? *Neurology* 65 1958–1960. 10.1212/01.wnl.0000188907.97339.76 16380619

[B26] Mustapic M. Eitan E. Werner J. K. Jr. Berkowitz S. T. Lazaropoulos M. P. Tran J. (2017). Plasma extracellular vesicles enriched for neuronal origin: a potential window into brain pathologic processes. *Front. Neurosci.* 11:278. 10.3389/fnins.2017.00278 28588440 PMC5439289

[B27] Ohmichi T. Mitsuhashi M. Tatebe H. Kasai T. Ali El-Agnaf O. M. Tokuda T. (2018). Quantification of brain-derived extracellular vesicles in plasma as a biomarker to diagnose Parkinson’s and related diseases. *Parkin. Relat. Disord.* 61 82–87. 10.1016/j.parkreldis.2018.11.021 30502924

[B28] Ono S. Hu J. Shimizu N. Imai T. Nakagawa H. (2001). Increased interleukin-6 of skin and serum in amyotrophic lateral sclerosis. *J. Neurol. Sci.* 187 27–34. 10.1016/s0022-510x(01)00514-7 11440741

[B29] Pronto-Laborinho A. Pinto S. Gromicho M. Pereira M. Swash M. de Carvalho M. (2019). Interleukin-6 and amyotrophic lateral sclerosis. *J. Neurol. Sci.* 398 50–53. 10.1016/j.jns.2019.01.026 30682521

[B30] Raposo G. Stoorvogel W. (2013). Extracellular vesicles: exosomes, microvesicles, and friends. *J. Cell. Biol.* 200 373–383. 10.1083/jcb.201211138 23420871 PMC3575529

[B31] Rothstein J. D. (2017). Edaravone: a new drug approved for ALS. *Cell* 171:725. 10.1016/j.cell.2017.10.011 29100067

[B32] Sekizawa T. Openshaw H. Ohbo K. Sugamura K. Itoyama Y. Niland J. C. (1998). Cerebrospinal fluid interleukin 6 in amyotrophic lateral sclerosis: immunological parameter and comparison with inflammatory and non-inflammatory central nervous system diseases. *J. Neurol. Sci.* 154 194–199. 10.1016/s0022-510x(97)00228-1 9562310

[B33] Spooren A. Kolmus K. Laureys G. Clinckers R. De Keyser J. Haegeman G. (2011). Interleukin-6, a mental cytokine. *Brain Res. Rev.* 67 157–183. 10.1016/j.brainresrev.2011.01.002 21238488

[B34] Su X. W. Simmons Z. Mitchell R. M. Kong L. Stephens H. E. Connor J. R. (2013). Biomarker-based predictive models for prognosis in amyotrophic lateral sclerosis. *JAMA Neurol.* 70 1505–1511. 10.1001/jamaneurol.2013.4646 24145899

[B35] Tanaka M. Kikuchi H. Ishizu T. Minohara M. Osoegawa M. Motomura K. (2006). Intrathecal upregulation of granulocyte colony stimulating factor and its neuroprotective actions on motor neurons in amyotrophic lateral sclerosis. *J. Neuropathol. Exp. Neurol.* 65 816–825. 10.1097/01.jnen.0000232025.84238.e1 16896315

[B36] Van Wagoner N. J. Benveniste E. N. (1999). Interleukin-6 expression and regulation in astrocytes. *J. Neuroimmunol.* 100 124–139. 10.1016/s0165-5728(99)00187-3 10695723

[B37] Vu L. T. Bowser R. (2017). Fluid-Based biomarkers for amyotrophic lateral sclerosis. *Neurotherapeutics* 14 119–134. 10.1007/s13311-016-0503-x 27933485 PMC5233638

[B38] Winston C. N. Goetzl E. J. Akers J. C. Carter B. S. Rockenstein E. M. Galasko D. (2016). Prediction of conversion from mild cognitive impairment to dementia with neuronally derived blood exosome protein profile. *Alzheimers Dement* 3 63–72. 10.1016/j.dadm.2016.04.001 27408937 PMC4925777

[B39] Yanez-Mo M. Siljander P. R. Andreu Z. Zavec A. B. Borras F. E. Buzas E. I. (2015). Biological properties of extracellular vesicles and their physiological functions. *J. Extracell. Vesicles* 4:27066. 10.3402/jev.v4.27066 25979354 PMC4433489

[B40] Zhao W. Beers D. R. Appel S. H. (2013). Immune-mediated mechanisms in the pathoprogression of amyotrophic lateral sclerosis. *J. Neuroimmune. Pharmacol.* 8 888–899. 10.1007/s11481-013-9489-x 23881705 PMC4126425

