# Supplementary material for: Increased Interleukin-6 Levels in the Astrocyte-Derived Exosomes of Sporadic Amyotrophic Lateral Sclerosis Patients
Source: Front Neurosci. 2019 Jun 5;13:574. doi: 10.3389/fnins.2019.00574 (PMC6560167; doi:10.3389/fnins.2019.00574)
Supplement: Supplementary file 1 [file Data_Sheet_1.PDF]

**Supplementary material**

**Supplementary Method**

Western blot and transmission electron microscope analysis of ADEs

To do the western blot of astrocyte-derived exosomes, we have made some minor modifications to extract ADEs. Briefly, 2ml plasma from one subjects was used and divided into 4 samples to extract ADEs separately at the beginning. The 4 samples were combined to one sample before the elution step. The 3% bovine serum albumin (BSA) was replaced by the Phosphate-buffered Saline (0.1M phosphate, 0.15M NaCl; pH=7.2) in the whole step. Before the elution step, the streptavidin-agarose Ultralink resin were washed with the Phosphate-buffered Saline added with 0.1% SDS for 5 times. In the elution step, 150 ul SDS-PAGE sample buffer (2% SDS, 62.5mM Tris base, 10% glycerol, 2.5% 2-mercaptoethanol, pH=6.8) was added and the sample was be boiled. In the negative control group one, the biotinylated anti-ACSA-1 antibody was replaced with Phosphate-buffered Saline, not the 3% BSA. Prepared ADEs protein and plasma protein were separated on SDS-PAGE gel and transferred to nitrocellulose membranes. Membranes were blotted with antibodies to CD63 (1:1000; abcam), calnexin (1:1000, abcam) and IgG (1:500, Bioss), then incubated with appropriate HRP-conjugated secondary antibodies and visualized by enhanced chemiluminescence (Pierce).

For electron microscopy analysis, ADEs were eluted with 100 µl cold 0.05 M glycine-HCl (pH=3.0) and neutralized subsequently. 10 ul suspended ADEs were mounted onto copper grid, fixed with glutaraldehyde and stained with Uranium dioxide acetate, then analyzed under transmission electron microscope (JEOL-JEM1400).

**Supplementary Results:**

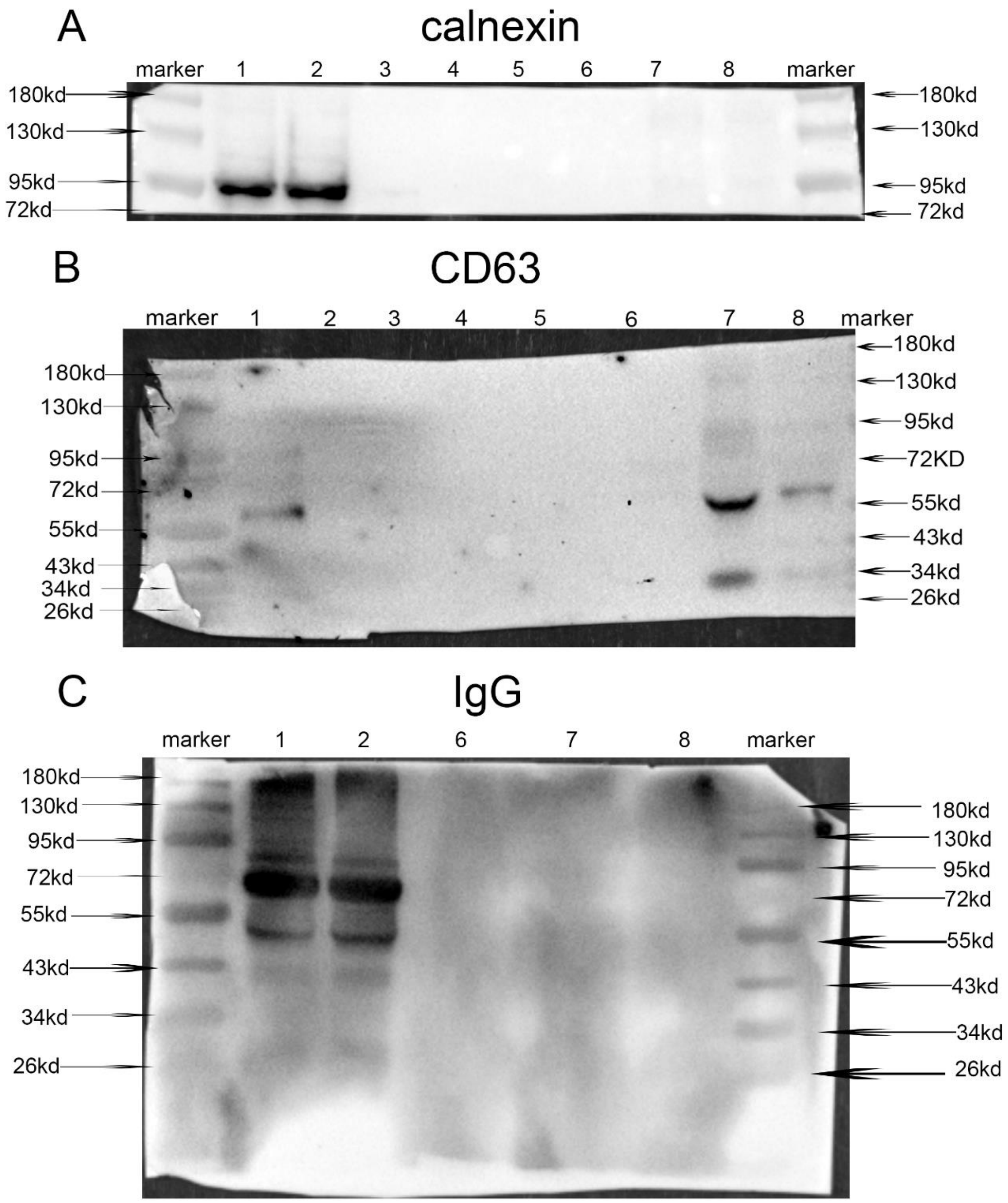


**Supplementary figure 1:** validate the ADEs by western blot. The ADEs were positive for CD63, but negative for calnexin and IgG. 1,2 indicate plasma; 3,4: negative control group two; 5,6: negative control group one;7,8: ADEs.

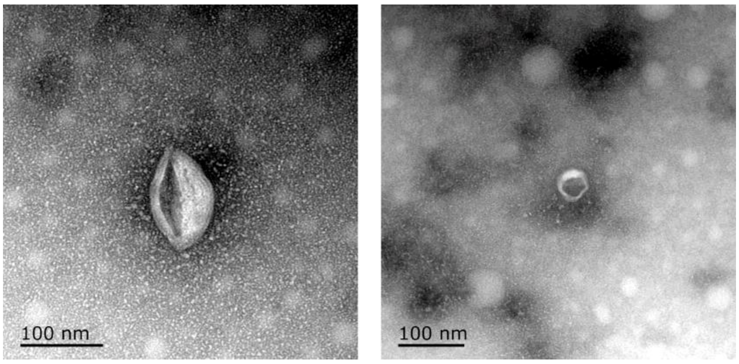


**Supplementary figure 2:** verify the ADEs by transmission electron microscope. Electron micrograph shows the morphology and size of exosomes.

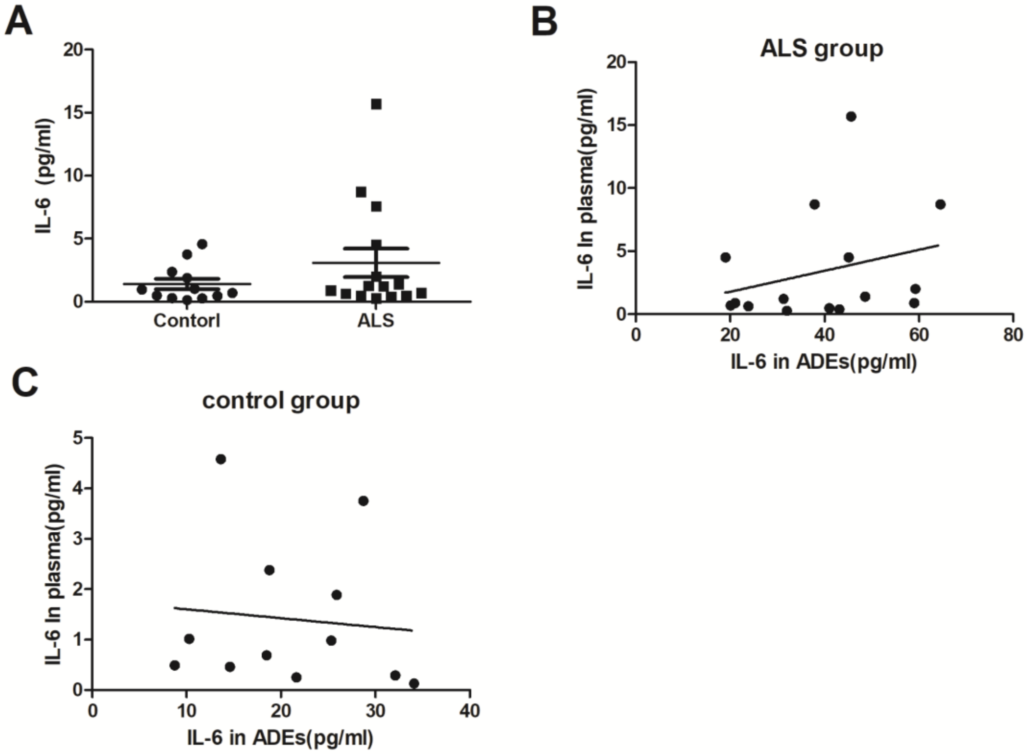


**Supplementary figure 3**: Comparison between plasma IL-6 levels in controls and ALS patients and correlations between IL-6 levels in plasma and ADEs. A: the IL-6 was detectable only in 12 controls and 15 ALS patients. The plasma IL-6 levels ranged from 0.13 to 4.58 pg/mL in controls and 0.39 to 15.69 pg/ml in ALS patients. There was no difference in plasma IL-6 levels between controls and ALS patients (*p*=0.3614). B,: no correlation between IL-6 levels in plasma and ADEs in ALS group(r=0.3384,*p*=0.2173); C: no correlation between IL-6 levels in plasma and ADEs in control group(r=-0.2657,*p*=0.4038).
